# Variation in Tracheid Dimensions of Conifer Xylem Reveals Evidence of Adaptation to Environmental Conditions

**DOI:** 10.3389/fpls.2022.774241

**Published:** 2022-02-17

**Authors:** Jingming Zheng, Yajin Li, Hugh Morris, Filip Vandelook, Steven Jansen

**Affiliations:** ^1^Beijing Key Laboratory for Forest Resources and Ecosystem Processes, School of Ecology and Nature Conservation, Beijing Forestry University, Beijing, China; ^2^Department of Integrative Biology and Biodiversity Research, Institute of Botany, University of Natural Resources and Life Sciences, Vienna, Austria; ^3^Living Collections Department, Meise Botanic Garden, Meise, Belgium; ^4^Institute of Systematic Botany and Ecology, Ulm University, Ulm, Germany

**Keywords:** xylem structure, tracheid diameter, tracheid wall thickness, latitude, climate, soil

## Abstract

Globally distributed extant conifer species must adapt to various environmental conditions, which would be reflected in their xylem structure, especially in the tracheid characteristics of earlywood and latewood. With an anatomical trait dataset of 78 conifer species growing throughout China, an interspecific study within a phylogenetic context was conducted to quantify variance of tracheid dimensions and their response to climatic and soil conditions. There was a significant difference in tracheid diameter between earlywood and latewood while no significant difference was detected in tracheid wall thickness through a phylogenetically paired *t*-test. Through a phylogenetic principle component analysis, Pinaceae species were found to be strongly divergent in their tracheid structure in contrast to a conservative tracheid structure in species of Cupressaceae, Taxaceae, and Podocarpaceae. Tracheid wall thickness decreased from high to low latitudes in both earlywood and latewood, with tracheid diameter decreasing for latewood only. According to the most parsimonious phylogenetic general least square models, environment and phylogeny together could explain about 21∼56% of tracheid structure variance. Our results provide insights into the effects of climate and soil on the xylem structure of conifer species thus furthering our understanding of the trees’ response to global change.

## Introduction

Understanding how trees respond to environmental conditions (e.g., climate, soil, etc.) is crucial for an accurate prediction of future changes to forest dynamics caused by global warming, especially in the Northern boreal ecosystems (Vaganov et al., 2006; Zhang et al., 2020). A latitudinal gradient is associated with consistent temperature differences that can act as a natural laboratory, helping us to understand forest responses to global warming (De Frenne et al., 2013). Although there are studies on tree growth response to latitude, most of them focus on the effects of climatic factors on ring width and wood density for a limited number of species (Wettstein et al., 2011; Björklund et al., 2017), while there are few inter-specific studies on the xylem anatomical response to various environmental conditions. Conduit dimensions of xylem have historically been important traits for both angiosperms and gymnosperms given their multiple functions vital to tree growth (Hacke, 2015). Similar to angiosperm vessels, tracheid dimensions can provide us with valuable information about how conifer species adapt to various environments and their possible responses to climate change (Vaganov et al., 2006). However, the latitudinal patterns of tracheid traits have been surprisingly understudied compared to vessel traits (Brodribb et al., 2012).

Conifer xylem has less cellular diversity than angiosperm xylem, and consists of two cell types: tracheids (approx. 90–93% of the xylem surface area) and parenchyma (approx. 7–10% surface area) (Hacke, 2015). Temperate conifer tree rings are mainly composed of two tracheid types: (1) large and thin-walled earlywood tracheids, which are produced early in the growing season and are primarily responsible for water transport, and (2) small but thick-walled latewood tracheid, which are produced after the earlywood cells and serving for mechanical support (Fonti et al., 2013). Therefore, tracheid diameter and wall thickness represent useful proxies to examine tracheid cell profiles within a growth ring and across xylem. This is especially true in studies using old trees as samples, since tracheid size tends to be constant as the tree ages (Vysotskaya and Vaganov, 1989; Zhou and Jiang, 1994; Vaganov et al., 2006). In recent years, quantitative wood anatomy (QWA) emerged as a growing field of dendrochronology that allows obtaining a large number of parameters of xylem cells and structure, highlighting the adjustments of trees to their environment and relationship between cell structure and functions (Arzac et al., 2021). Understanding the temporal pattern of these traits and how the environment shapes them are of high importance for forest management.

Tracheid cell development processes are influenced by environmental conditions, especially climatic conditions (Castagneri et al., 2017). Recent studies on wood formation dynamics provide some insights into the process of xylogenesis, i.e., xylem formation (Rathgeber et al., 2016). For instance, Rossi et al. (2016) found that the timing of xylem phenology events and mean annual temperature of different sites were related linearly for 10 pine species in the Northern Hemisphere. The latter authors suggested that the uniformity of the process of wood formation was mainly determined by the climatic conditions occurring at the time of growth resumption (Rossi et al., 2016). In short, cell enlargement and secondary cell wall deposition and lignification (wall thickening) are the two fundamental sub-processes of xylogenesis. The complex interplay between the duration and rate of xylogenesis determines the changes in tracheid dimensions, e.g., cell and lumen diameter, lumen area and wall thickness, which in-turn creates the anatomical structure driving the wood density profile (Cuny et al., 2014). So far, our understanding of how environmental conditions affect xylogenesis is still incomplete and many relevant results were often species-specific and site-dependent with a strong focus on temperature effects (Cuny et al., 2014; Rossi et al., 2016; Buttò et al., 2019). There are relatively few interspecific studies on the effect of other environmental factors on xylem anatomical traits. Furthermore, previous studies on tracheid dimensions generally focused on radial tracheid diameter and wall thickness within-rings (Vaganov et al., 2006). Including tangential tracheid diameter and wall thickness for various conifer species could provide more valuable information in a comparative study.

Although previous research has suggested that hydraulic traits like tracheid dimensions are correlated with both precipitation and temperature, temperature is being increasingly recognized as the primary driver of conifer growth reactivation in a cold climate. However, most studies have only focused on temperate and boreal ecosystems in which snowmelt provides abundant water especially at the beginning of spring and summer, and where water availability is not a limiting factor for conifer xylem formation (Cuny and Rathgeber, 2016; Rathgeber et al., 2016; Rossi et al., 2016). Given that xylem cell expansion is a turgor-driven process depending on cellular water uptake and solute accumulation, water availability can affect xylogenesis (Kozlowski and Pallardy, 2002). Researchers have shown that both cell division and expansion are sensitive to water potential (Fonti et al., 2010), and that water deficit is the primary constraint for xylogenesis of *Pinus pinaster* in a Mediterranean climate (Vieira and Campelo, 2020). A study on *Juniperus przewalskii* provides additional evidence that soil moisture is more important than temperature at initiating xylem growth under cold and dry conditions (Ren et al., 2018). These results suggest that wood formation and xylem structure of various conifers could be controlled by genetic and multiple environmental factors such as temperature, soil moisture content and other soil properties, thus an interspecific study in a phylogenetic context and along a wide latitudinal gradient could shed light on this important research topic.

Although conifer xylem mainly consists of tracheids, the distribution of extant conifer species is globally as wide as angiosperms (Yang et al., 2017). Meanwhile, boreal forests at high latitudinal regions consist mainly of a few conifer species whereas in low latitudinal regions most conifer species generally tend in inhabit mountainous regions. The latter indicates that conifer trees are more adapted to stresses (i.e., coldness and drought) than angiosperm trees, allowing them to thrive in habitats unsuitable for angiosperm trees (Brodribb et al., 2012). Moreover, the adaptation of conifers is due to differences in life history strategies between them and angiosperms, such as traits that confer greater resistance to freezing and drought events and a high nutrient use efficiency (Brodribb et al., 2012). Therefore, adaptation to environmental conditions would be reflected in their xylem structure, especially in tracheid dimensions in both earlywood and latewood due to construction cost and water transportation constraints in addition to highly specialized bordered pits (Bouche et al., 2014). As tracheids of earlywood and latewood function differently, we hypothesize that the xylem structure of conifer species is mainly a consequence of adaptation to cold and/or drought stress, and that differences in tracheid dimensions between earlywood and latewood could be viewed as an ecological strategy to changing environmental conditions along a latitudinal gradient, especially temperature and precipitation. Specifically, we address the following questions: (1) do tracheid dimensions always differ between earlywood and latewood? (2) What is the role of phylogeny in associations among tracheid dimensions? (3) Are there clear trends of xylem structure along a latitudinal gradient? (4) How much of the xylem structure variance can be explained by environment and phylogeny, respectively? To the best of our knowledge, there is currently no cross-species anatomical study of conifers along such a wide gradient, making our study an important contribution to plant science.

## Materials and Methods

### Data Collection

China is among the countries with the highest number of conifer species in the world and with more than 90 species being endemics (Farjon, 2017; Yang et al., 2017). Meanwhile, the land mass of China comprises a large latitudinal gradient from 18° N to 54 ° N and longitudinal gradient from 73 ° E to 135 ° E in tandem with various topological, climatic and soil conditions. Of note, the climate in China is a typical temperate monsoonal climate, which is characterized by synchrony of temperature and precipitation, i.e., obvious climatic seasonality (Ding et al., 2013). Such diverse species and environmental conditions provide an opportunity for an interspecific investigation on the correlation between xylem structures and environmental (i.e., climate and soil) conditions.

**FIGURE 1:**
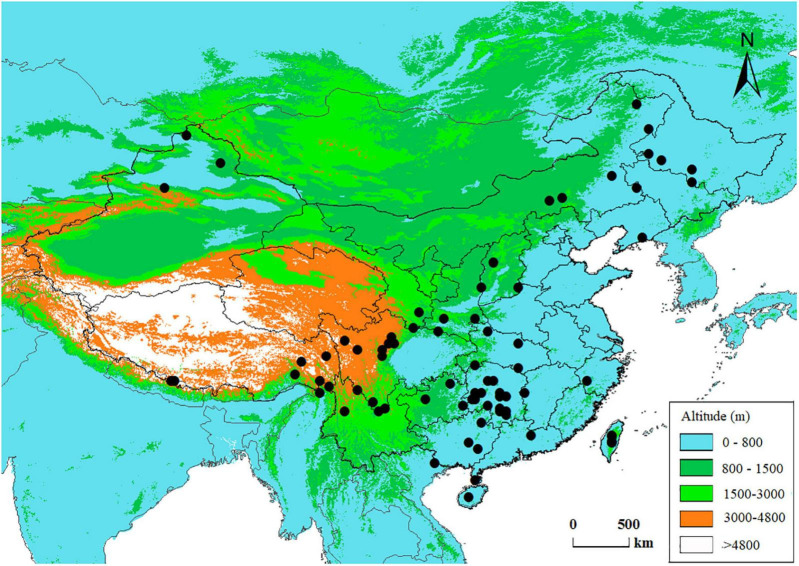
Spatial distribution of the data in this study. Each dot represents a species’ central distribution location on land of China, using the mid-latitude and mid-longitude values of the species’ range in the country as coordinates.

We compiled an anatomical dataset for Chinese conifer species including tracheid characters from both earlywood and latewood. The anatomical dataset is a product of a nation-wide research program on wood properties carried out in the 1990s, during which stem wood samples for conifer and broad-leaved tree species across China were collected according to a national standard GB 1927–1991 (Chinese Ministry of Forestry, 1991; Yang et al., 2001). The wood samples were maintained in the wood collections of the Chinese Academy of Forestry, and the wood anatomical traits were measured following consistent methods (Zhou and Jiang, 1994). Taxonomic names were verified using the Plant List^1^ to correct for synonyms. Varieties and subspecies were removed since the study is at an interspecific level. Finally, we obtained wood anatomy data from 78 conifer species and *Ginkgo biloba* (Ginkgoales), which are distributed across China (Figure 1 and Supplementary Table 1). *Ginkgo biloba* L. is a relic non-coniferous gymnosperm species native to China, we included this species as an outgroup in the phylogeny of gymnospermae in this phylogenetic comparative study.

Tracheid dimensional traits include tracheid diameter and tracheid wall thickness measured from tangential and radial directions for earlywood and latewood, respectively (Supplementary Figure 1). Tracheid diameter is the distance between outer boundaries of a tracheid cell while tracheid wall thickness is single cell wall thickness of a tracheid cell with tracheid corners not included. Finally, eight traits were selected from the dataset in this study (Table 1), including tangential and radial tracheid diameter for earlywood (CTD.e and CRD.e) and latewood (CTD.l and CRD.l), and tangential and radial tracheid wall thicknesses for earlywood (WTT.e and WRT.e) and latewood (WTT.l and WRT.l). All anatomical observations were limited to wood samples from one individual per species, assuming that intraspecific variation was smaller than interspecific (Morris et al., 2016). The wood samples were taken from mature trees with a DBH over 20 cm in natural forests for previous studies suggested that tracheid size is consistent throughout sapwood of older specimens (Zhou and Jiang, 1994). The sampled disks were collected at a height of 1.3 m. These disks were cut, from pith to bark, into six equal parts according to the equidistance method and anatomical samples were taken from the middle of the outermost part (the closest to the bark), avoiding reaction wood. The entire span of one growth ring was sectioned with a microtome and wood anatomical traits were measured using a light microscope for earlywood and latewood, respectively. The earlywood and latewood subzones were chosen as close as possible to the ring boundaries as seen under a microscope and tracheids at the very end of latewood with irregular shape were avoided. For tracheid diameter and tracheid wall thickness, 100 tracheids were randomly measured in earlywood and latewood subsection for each sample (Yang et al., 2001).

**TABLE 1:** Traits and environmental factors examined in this study, with reference to their acronyms, units, and definition.

	Units	Description
**Geography gradient**		
Midpoint latitude (LAT)		Midpoint latitude of a species’ distribution area
Midpoint longitude (LON)		Midpoint longitude of a species’ distribution area
Midpoint altitude (ALT)	m	Midpoint altitude of a species’ distribution area
**Tracheid traits**		
Cell tangential diameter for earlywood tracheids (CTD.e)	μm	Tracheid diameter in tangential direction in the cross section of a wood slide for earlywood subzone
Cell tangential diameter for latewood tracheids (CTD.l)	μm	Tracheid diameter in tangential direction in the cross section of a wood slide for latewood subzone
Cell radial diameter for earlywood tracheids (CRD.e)	μm	Tracheid diameter in radial direction in the cross section of a wood slide for earlywood subzone
Cell radial diameter for latewood tracheids (CRD.l)	μm	Tracheid diameter in radial direction in the cross section of a wood slide for latewood subzone
Cell wall tangential thickness for earlywood tracheids (WTT.e)	μm	Tracheid cell wall thickness in tangential direction in the cross section of a wood slide for earlywood subzone
Cell wall tangential thickness for latewood tracheids (WTT.l)	μm	Tracheid cell wall thickness in tangential direction in the cross section of a wood slide for latewood subzone
Cell wall radial thickness for earlywood tracheids (WRT.e)	μm	Tracheid cell wall thickness in radial direction in the cross section of a wood slide for earlywood subzone
Cell wall radial thickness for latewood tracheids (WRT.l)	μm	Tracheid cell wall thickness in radial direction in the cross section of a wood slide for latewood subzone
Maximum plant height (H_*max*_)	m	Maximum height of a plant species
**Climatic indices**		
Mean annual temperature (MAT)	°C	Mean annual temperature
Mean annual precipitation (MAP)	mm	Mean annual precipitation
Temperature seasonality (TSEA)		The standard deviation of monthly temperature
Precipitation seasonality (PSEA)		The coefficient of monthly precipitation variance
**Soil indices**		
pH index (PH)		pH index in 30∼60 cm soil depth (H_2_O solution)
Coarse fragment (CFVO)	cm^3^/cm^3^	Coarse fragment in 30∼60 cm soil depth (volumetric)
Sand content (SAND)	kg/kg	Sand content in 30∼60 cm soil depth (gravimetric)
Silt content (SILT)	kg/kg	Silt content in 30∼60 cm soil depth (gravimetric)
Clay content (CLAY)	kg/kg	Clay content in 30∼60 cm soil depth (gravimetric)

The tracheid traits for each species were measured from wood sample of the same individual tree, which makes the data ideal for comparative analyses. However, this may lead to uncertainty of intraspecific variance for the anatomical traits due to lack of replicates. To validate the dataset, we compiled the average values of tracheid diameter and wall thickness of earlywood and latewood from the Atlas of Gymnosperms Woods of China (Jiang et al., 2010). This book consists of anatomical descriptions of 140 conifer species in China, but only provides average tracheid values from multiple tree samples for each species across its natural range. We compared the data from Jiang et al. (2010) with the tracheid traits we measured from tangential and radial directions, respectively. Finally, we extracted average dimensional value for the shared conifer species with current dataset for its validation (Supplementary Table 1). Correlations of corresponding traits in the two data sources were all highly significant (*p* < 0.01), suggesting that intraspecific variance is of minor importance in our dataset. For example, the correlation coefficients between average diameter for earlywood tracheid from the Wood Atlas book and CTD.e and CRD.e in our dataset were 0.64 and 0.77, respectively (*p* < 0.01), while the coefficients between average wall thickness for latewood from the Atlas book vs. WTT.l and WRT.l were 0.61 and 0.63, respectively (*p* < 0.01). In addition, the maximum plant height for each species was extracted from the Chinese Higher Plants (Fu, 2012) to check their potential relationships with conduit size (Olson et al., 2021).

Locations and environmental information for each species were needed to explore latitudinal patterns of tracheid traits and their environmental correlations. As the sampling locations were unfortunately not recorded for most of the species in the database, coordinates data for each species’ distribution within China were retrieved from the Atlas of the Gymnosperms of China (Ying et al., 2004), which is comparable to retrieving GBIF data by species names for large scale pattern analysis in ecological studies (García-Roselló et al., 2015; Morris et al., 2016). Using 4766 retrieved coordinates, we extracted environmental indices including altitude from SRTM^2^, climatic variables from WorldClim^3^ (Ficka and Hijmans, 2017), and soil properties from SoilGrids^4^ (Hengl et al., 2014). A total of 12 environmental variables were chosen given that temperate monsoonal climate dominates the study area (Table 1), including: (1) Geographic indices: latitude (LAT, °), longitude (LON, °), and altitude (ALT, m). (2) Climatic indices: mean annual temperature (MAT, °C), temperature seasonality (TSEA, the standard deviation of monthly temperature), mean annual precipitation (MAP, mm), and precipitation seasonality (PSEA, the coefficient of monthly precipitation variance). (3) Soil indices: pH index (pH), coarse fragment (CFVO, cm^3^/cm^3^), sand content (SAND, kg/kg), silt content (SILT, kg/kg), and clay content (CLAY, kg/kg). Median values of environmental variables from each species distribution range were used as environmental variables for the species.

Previous studies demonstrated that xylogenesis and the resulting xylem structure were species-specific (Rathgeber et al., 2016; Chen et al., 2019). Therefore, our study factored in phylogeny using various phylogenetic models. We firstly constructed a phylogenetic tree for the species using the largest dated vascular plant phylogeny presently available, the GBOTB mega-tree of Smith and Brown (2018), which includes 79,881 taxa and all families of extant vascular plants based on combined molecular data from GenBank and data from the Open Tree of Life project (Smith and Brown, 2018). We then pruned the tree using the “phylo.maker” function in R package “V.PhyloMaker” (Jin and Qian, 2019), to generate a phylogeny for the 79 species for phylogenetic analyses.

### Data Analysis

Statistics and phylogenetic modeling were conducted using R (R Core Team, 2018). Phylogenetic signals for each tracheid trait were estimated using the “phylosig” function in the package “phytools” (Revell, 2012). Phylogenetic independent contrast (PICs) of tracheid characters and maximum plant height were calculated using the “pic” function in the package “ape” (Paradis and Schliep, 2018), and their correlations were tested using the “corr.test” function in package “psych” (Revelle, 2018). However, since PICs of maximum plant height were not related to any of the tracheid traits (*p* > 0.05, Supplementary Table 3), the maximum plant height was excluded in later analyses. A phylogenetically paired *T*-test was performed to test for significant differences between corresponding traits in earlywood and latewood using the “phyl.pairedttest” function in the package “phytools” (Revell, 2012), which is similar to a paired *t*-test but takes phylogeny into account (Supplementary Table 2).

A phylogenetic generalized least square model (PGLS) was used to build univariate and multi-variate using the “gls” function in the package “nlme” (Pinheiro et al., 2015). First, univariate models for each trait were fitted as a function of each environmental index, and R^2^ for each model was calculated using the “R2.pred” function in package “rr2,” which further partitioned the R^2^ into contributions of phylogeny and environmental variables (Ives, 2019). Specially, latitudinal patterns that existed for each trait were tested using univariate PGLS models with midpoint latitude as a predictor. Second, multi-variate models for each trait were fitted as functions of climatic and soil indices since most climatic variables and soil variables were related to latitude (Supplementary Figure 2). To reduce the number of predictors to minimize collinearity, we only included environmental variables that were significant in univariate models, and their possible combinations, in order to build a series of models. We did not include any interactions to avoid overwhelming the available degrees of freedom in the models. For each group of multivariate models, the most parsimonious models were selected based on lowest Akaike Information Criterion (AIC) value and *R*^2^ was partitioned in the same way as the aforementioned.

As most of the eight tracheid traits were positively correlated, a principal component analysis (PCA) was carried out to reduce the tracheid traits into principle components (PCs) using the “princomp” function in the package “stats”, and PC1 and PC2 were used as two new traits depicting the whole xylem character in building PGLS models. Meanwhile, a phylogenetic principal component analysis (pPCA) was employed to detect non-independent values of variables (PCs) with the phylogenetic relationship between species (phylogenetic autocorrelation) using the “ppca” function in package “adephylo” (Jombart et al., 2010). In our pPCA, phylogenetic proximities were calculated using Abouheif’s proximity, and the resulting matrix of phylogenetic proximities was used to calculate phylogenetic autocorrelation, i.e., *Moran’s I* value. A negative autocorrelation was a result of differences among closely related species in the tips of the phylogeny while a positive autocorrelation was a result of similarity in related species in the tips of the phylogeny (Jombart et al., 2010).

## Results

### Difference of Tracheid Traits Between Earlywood and Latewood

**FIGURE 2:**
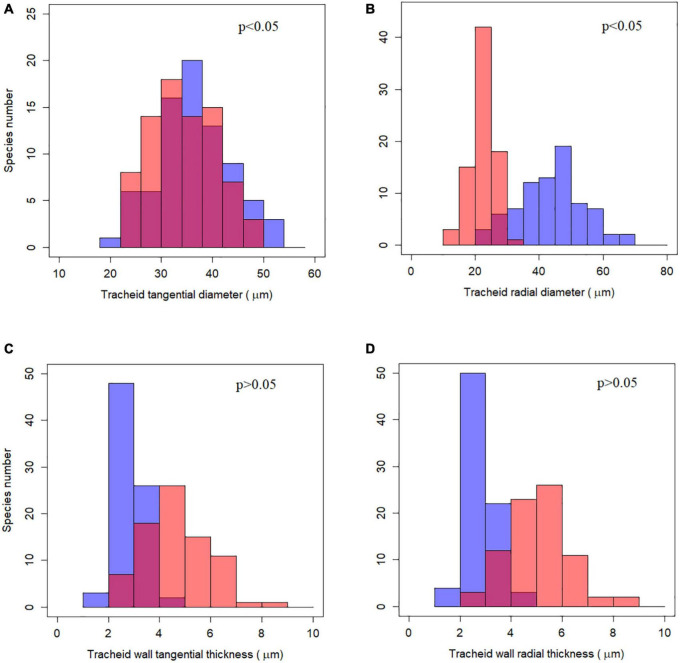
Frequency distribution of tracheid traits and their phylogenetically paired *t*-test between earlywood and latewood. **(A–D)** Represent the four tracheid traits studied respectively. Blue bars stand for earlywood and red bars for latewood, purple color indicates overlaying of blue and red bars. The *p*-value for phylogenetically paired *t*-test is shown at the up-right corner, indicating that tracheid diameters between earlywood and latewood were significantly different while tracheid wall thickness was not.

Although tracheid diameter and wall thickness differed significantly between earlywood and latewood using a non-phylogenetic paired *t*-test (*p* < 0.001), the relationships did not hold true when taking phylogeny into account (Figure 2 and Supplementary Table 2). The two indices of tracheid diameter still differed for earlywood and latewood under phylogenetically paired *t*-test (*p* < 0.05) while tracheid wall thickness was not significantly different (*p* > 0.05).

### Associations Among Tracheid Traits

**FIGURE 3:**
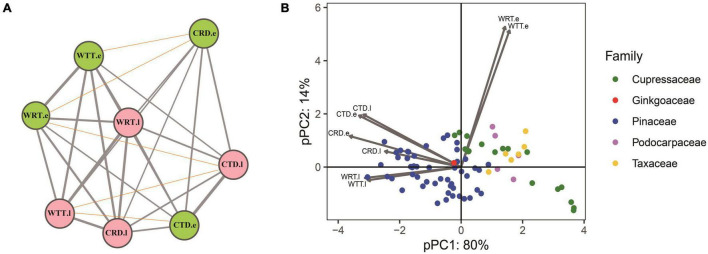
Results of a correlation analysis and principle component analysis for tracheid traits in a phylogenetic context. **(A)** Correlations among PICs of the eight tracheid traits. Pink and green colors stand for earlywood and latewood, respectively, the width of gray lines stands for positive correlation coefficients between two traits, while red thin lines stand for non-significant correlations. **(B)** pPCA of eight tracheid traits for conifers species from 5 families, showing that Pinaceae species and Cupressaceae species take up a different position along the first PC axis. Acronyms of xylem traits and environmental variables are same in Table 1.

Among 31 correlation tests for the PICs of the eight traits, 26 of them were positively correlated (*P* < 0.05) while five did not show significant correlations (*p* < 0.05), and no negative correlations were found (Figure 3A). Results of the pPCA showed that PC1 contains more than 80% information from the tracheid traits measured, thus PC1 could be regarded as representing major characteristics of the tracheid structure. Among the 78 conifers belonging to four families, Pinaceae and Cupressaceae species were at opposite positions along the PC1 axes (Figure 3B). Species of Taxaceae and Podocarpaceae were also at the opposite position to Pinaceae species but this was difficult to confirm due to their low species number in our study.

### Divergence of Tracheid Traits Along Phylogeny

**FIGURE 4:**
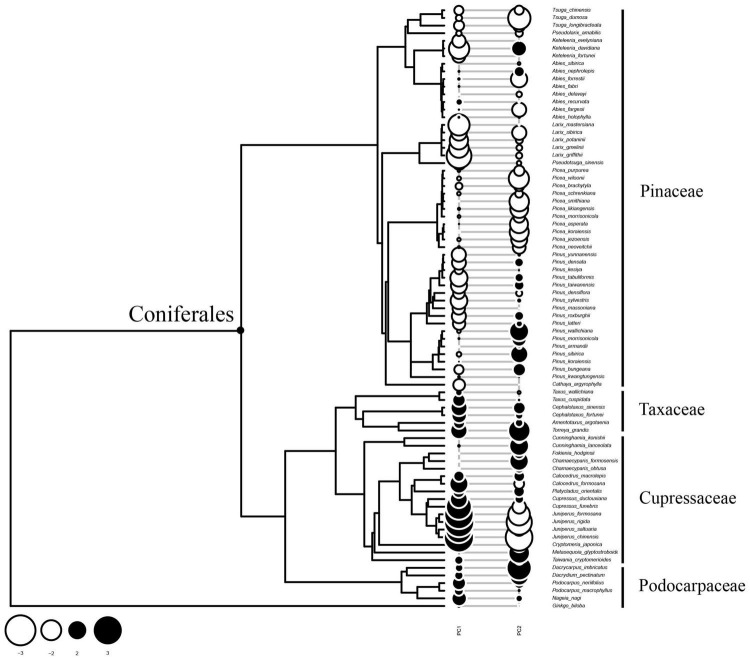
Pattern of phylogenetic autocorrelation of tracheid dimensions along the phylotree. A bubble stands for *Moran’ I* values for each species. A coniferales label was added to emphasize two clades: Pinaceae species at the upper part of the phylotree and species of other families at the lower branches. Most of Pinaceae species showed negative phylogenetic autocorrelation values, suggesting that there are more frequent divergences in tracheid traits related with PC1. On the contrary, most of Cupressaceae species and others showed positive values, suggesting xylem structure were conserved for these species.

Since PC1 determined to a large extent the tracheid features measured, the conifer species studied were divided into two groups along the phylotree (Figure 4). Pinaceae species were located at the upper part of the phylotree and showed mainly negative Moran’s *I* values (negative autocorrelations along phylogeny), which was especially the case for *Larix* spp., *Keteleeria* spp., and some *Pinus* spp. However, species of Cupressaceae and another two families at the lower part of the phylotree showed mainly positive Moran’ values (positive autocorrelations along phylogeny), especially in *Juniperus* spp. and *Cupressus* spp. This pattern suggested that tracheid dimensions diverged strongly for Pinaceae species, but was generally conserved in Cupressaceae, Podocarpaceae, and Taxaceae.

### Latitudinal Patterns of Tracheid Features in Conifer Species

**FIGURE 5:**
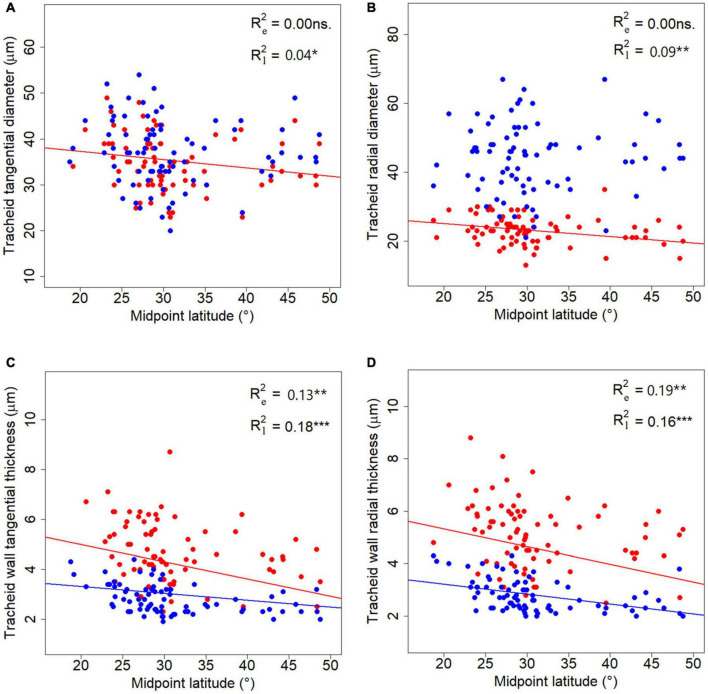
Latitudinal trends of tracheid traits. **(A–D)** Represent latitudinal trends for the four tracheid traits studied respectively. Blue and red lines stand for fitted GPLS models for earlywood and latewood data, respectively. *R*^2^ stands for the explanatory power of the latitude alone in the PGLS models, subscript “e” in *R*^2^ stands for earlywood, while “l” stands for latewood. “*”, “**”, and “***” represent significance at level of 0.05, 0.01, and 0.001 respectively.

No significant correlation was found between tracheid traits and longitude (LON) or altitude (ALT) (*p* > 0.05), although all traits had phylogenetic signals (Supplementary Table 3). Six tracheid traits, i.e., CTD and CRD for latewood, WTT and WRT for both earlywood and latewood, showed a latitudinal pattern in a phylogenetic context (Figure 5). Among the six traits measured, latewood traits demonstrated steeper declining trends along latitude compared to the corresponding earlywood traits. As a special case, phylogeny did not contribute to the radial tracheid wall thickness for earlywood (WRT) in the corresponding GPLS model (Figure 5D and Supplementary Table 3).

### Environmental Effects on Tracheid Characteristics

Climatic indices (especially MAT, MAP, and TSEA) and soil properties (especially PH, SILT, and CLAY) correlated with most of tracheid traits in the univariate models (Supplementary Table 3). The most parsimonious multivariate PGLS models with the environmental factors as predictors could explain 21∼55% variance in tracheid traits, suggesting that phylogeny and environment played a role in shaping tracheid dimensions (Table 2 and Supplementary Table 4). For the six tracheid traits with latitudinal trend (Figure 5), the combination of climate and soil contributed to their variance, with climate dominating the effect. For tangential and radial tracheid diameters (CTD.e and CRD.e) without obvious latitudinal trend, soil factors contributed the most to their variance. In the PC1 and PC2 models, environmental factors alone could explain 23 and 19% of the xylem variance, respectively, in contrast to phylogeny alone, which contributed 43 and 27% to the xylem variance.

**TABLE 2:** The most parsimonious PGLS models of xylem characters as functions of environmental indices with models’ *R*^2^ partitioned.

Model formula	Lambda	AIC	*R* ^2^_*tot*_	*R* ^2^_*var*_	*R* ^2^_*phy*_	*R* ^2^_*sha*_
CTD.e∼gls(SILT)	0.86	500.34	0.47	0.14	0.39	0.06
CTD.l∼gls(MAT+SILT)	0.82	481.29	0.48	0.18	0.31	0.01
CRD.e∼gls(PH+SILT)	0.93	545.86	0.55	0.10	0.50	0.05
CRD.l∼gls(TSEA+CLAY)	0.51	437.06	0.21	0.10	0.13	0.01
WTT.e∼gls(MAT+SILT)	0.76	121.05	0.34	0.22	0.16	0.04
WTT.l∼gls(MAP+SILT)	0.83	224.75	0.48	0.25	0.31	0.08
WRT.e∼gls(MAT+SILT)	0.00	124.47	0.32	0.32	0.00	0.00
WRT.l∼gls(MAP+SILT)	0.85	219.24	0.52	0.30	0.32	0.10
PC1∼gls(MAP+SILT)	0.91	304.82	0.56	0.23	0.43	0.10
PC2∼gls(MAT+SILT)	0.69	248.35	0.40	0.19	0.27	0.05

*Subscript “tot,” “var,” “phy,” and “sha” following R^2^ stand for R^2^ of the whole model, environmental variables alone, phylogeny alone, and shared part between environmental variables and phylogeny, respectively. Acronyms of xylem traits and environmental variables are same in Table 1. PC1 and PC2: the first and second principle components of the tracheid traits.*

## Discussion

Our results show that conifers adapt to their environmental conditions through changes in their xylem structure. Tracheid diameter and tracheid wall thickness for latewood decreased with a rise in latitude. Tracheid wall thickness, but not diameter for earlywood, also showed a decreasing latitudinal trend. Climatic condition and soil properties contributed to this latitudinal pattern, which explains about half of the variance for tracheid diameter and wall thickness. Meanwhile, phylogeny was found to play an important role in variance of conifer xylem structure, with Pinaceae species showing strong divergence in contrast to the conservatism of species from Cupressaceae, Podocarpaceae and Taxaceae. Our comparative study of 78 conifer species provides evidence of xylem adaptation to environmental conditions when factoring for phylogeny.

There is a widely held idea that earlywood is composed of wide, thin-walled tracheids in contrast to the narrow thick-walled tracheids of latewood. Through a phylogenetic paired *t*-test, our results showed that tracheid diameter in earlywood was larger than in latewood (Figures 2B,C and Supplementary Table 4), which partly supports the latter idea. An explanation could be that the duration of latewood tracheid development was shorter than for earlywood, while a large proportion of carbohydrates produced in the previous and current growing season had been consumed by earlywood cell wall thickening (Kagawa et al., 2006; Rathgeber et al., 2016). Since the principal function of tracheids in earlywood is water transport, in contrast to latewood, which functions more in mechanical support and water storage (Domec and Gartner, 2002), the xylem structure presented by the tracheid diameter in earlywood and latewood were consistent with their functions. A large tracheid size in earlywood results in a large lumen diameter, enabling higher water conductivity while also increasing the risk of hydraulic failure associated with freezing-induced embolism. By contrast, a small tracheid diameter (<30 μm) is assumed to protect against freezing-induced embolism for conifers at high latitudes or altitudes (Pittermann and Sperry, 2003).

Contrary to tracheid diameter, the difference in tracheid wall thickness between earlywood and latewood was insignificant when taking phylogeny into account (Figures 2C,D and Supplementary Table 4). A possible explanation for our result was that the difference in tracheid wall thickness between earlywood and latewood was species-specific and may not always manifest the same pattern for numerous conifers. Although conifer species in boreal forests may have thicker cell walls in latewood compared to earlywood, conifer species in warm and wet regions, e.g., species of Podocarpaceae and Araucariaceae in the Southern Hemisphere, may not behave in the same way (Carlquist, 2017). Previous studies also found that tracheid wall thickness in intra-ring cells was surprisingly constant for some Pinaceae species in temperate regions (Cuny et al., 2014). However, the majority of conifer species are distributed throughout subtropical climates in our study (Figure 1), which may explain the insignificant differences in tracheid wall thickness between earlywood and latewood. From a functional point of view, mechanical strength enhancement of latewood tracheids was achieved not by cell wall thickness but due to the increased wall-to-range ratio from reduced tracheid diameter, especially in the radial direction (Pittermann et al., 2006).

Although many anatomical traits of woody angiosperms have shown a latitudinal trend (Wheeler et al., 2007; Zheng et al., 2019), studies showing similar results on gymnosperm wood have been less abundant until recently (Rossi et al., 2016; Björklund et al., 2017). In our cross-species analyses, we revealed a decrease in tracheid wall thickness for both earlywood and latewood together with tracheid diameter in latewood along a latitudinal gradient in China (Figure 3). In such a large latitudinal gradient under a monsoonal climate, the carbon investment for conifer xylem formation would decrease in the northern regions owing to a shortened photoperiod and a lower temperature during the growing season, resulting in a reduction in tracheid diameter and tracheid wall thickness in latewood. The latter finding is in good agreement with the fact that tree growth declines toward high latitudes. Besides, tracheid radial diameters of latewood were generally lower than 30 μm (Figure 5B), which is consistent with the threshold diameter against freezing-induced embolism (Sperry et al., 1994; Pittermann and Sperry, 2003). Therefore, the decreased stem hydraulic capacity in a cold region might be a consequence of the evolution of reduced vulnerability to freezing and drought-induced embolism, representing an ecological strategy for conifers distributed across colder regions (Creese et al., 2011). For instance, the wide distribution of *Pinus* species in the Northern Hemisphere was thought to be due to adaptation to cold temperature during the Eocene (Millar, 1993).

Climatic conditions (i.e., MAT, MAP, and TSEA) were correlated with different tracheid traits to varying degrees (Supplementary Table 3), which was largely in line with findings that climate could strongly control wood anatomy and formation (Pandey, 2021). Previous studies on boreal conifers showed that the period of wood formation lengthened linearly with MAT in a range of 14°C and contributed to increased tracheid dimensions for 10 conifer species in the Northern Hemisphere (Rossi et al., 2013). However, our results showed that temperature, precipitation and temperature seasonality all contributed to lumen diameter and tracheid wall dimensions for multiple conifer species across various climate conditions (i.e., temperate, subtropical, and tropical climate) after taking phylogeny into account. We are aware of the limitations of the climate data extracted from WorldClim, as we could not analyze the effect of climatic conditions on wood formation for each location without introducing large bias. In addition, soil properties (i.e., PH, SILT, and CLAY) could explain part of the variance in tracheid traits, especially for tracheid diameter in earlywood that did not demonstrate a latitudinal trend (Table 2). Although conifers are largely confined to high latitudes and elevations or nutrient-poor soils, some conifer species nearly always grow better on more fertile soils than on infertile soils (Bond, 1989; Beets et al., 2001). A possible explanation for the role of soil on tracheid dimensions was that a high soil fertility could increase the growth rate of conifers, resulting in a larger tracheid diameter and thinner tracheid wall thickness (Bergh et al., 1999). As there are only a few studies on the effect of soil condition on tracheid dimensions compared to growth rate or wood density, how the interaction of soil and climate influences wood anatomy requires further investigation.

**FIGURE 6:**
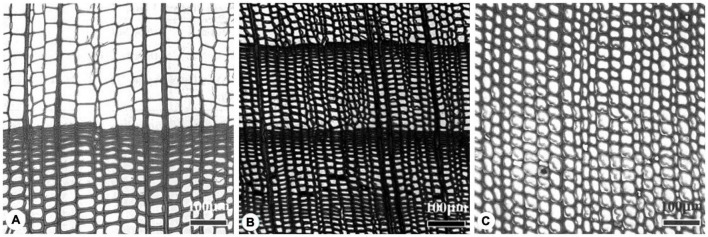
Cross-sectional images of various xylem structures from three species in different families. **(A)** *Abies holophylla* (Pinaceae) inhabits a high latitude region and is adapted to freezing events; its xylem consists of highly contrasting tracheid sizes between earlywood and latewood; **(B)** *Juniperus formosana* (Cupressaceae) inhabits a middle latitude region and is more adapted to drought; its xylem consists of relatively small tracheid sizes with a noticeable contrast between earlywood and latewood due to a dry season; **(C)** *Dacrydium pierrei* (Podocarpaceae) does not possess a clear growth ring boundary or variable tracheid size, a consequence of its distribution in low latitude regions where it competes for light with many angiosperm trees in the tropical forest. Furthermore, these three species also have distinct differences in leaf shapes and pit membrane morphology, which allude to the different evolutionary directions of the three species. The scale bars are all 100 μm in each image and the images were cited from Jiang et al. (2010) with modifications.

Xylem structure is a canvas for the evolutionary strategies of opposing functions through the production of two kinds of tracheid cells each year (Carlquist, 2017). Previous studies on conifer species from Pinaceae (Martínez-Vilalta et al., 2004), Cupressaceae (Pittermann et al., 2012), and Podocarpaceae (Turner and Cernusak, 2011) suggested that there are at least three evolutionary directions for conifer xylem adapting to different stresses in a geological time scale: cold adaptation, drought adaptation, and shade adaptation (Figure 6). For instance, both Cupressaceae spp. and Pinaceae spp. mainly grow in temperate climates. Cupressaceae are often restricted to more stressful cold and dry environments and show higher resistance to drought damage than coexisting Pinaceae. In a previous study on two coexisting conifers, *Pinus halepensis* and *Juniperus thurifera*, growing in a continental Mediterranean climate, it was shown that the two species significantly differ in the wood-anatomical features, such as distribution of tracheid lumen area and cell wall thickness (Pacheco et al., 2015). The authors explained their different vulnerability to drought-induced die-off by the fact that Cupressaceae have evolved toward a drought resistant xylem, while Pinaceae evolved more toward cold resistant xylem. However, since Cupressaceae and Pinaceae are temperate conifer families, including many species with varying capacity of drought and cold resistance, comparative studies between the two families are rare. A similar situation also applies to the tropical conifer families Podocarpaceae and Araucariaceae (Zimmer et al., 2015).

During the evolution of conifers, selection acted to optimize xylem structure to fulfill both safety and efficiency of water transport (Hacke, 2015). Conifers must adapt to diverse and often difficult environments by adjusting their xylem structure at different levels from micro-to-macro, e.g., the pit structure at the pit level (Bouche et al., 2014) through to tracheid dimensions at the cell level (Pittermann et al., 2006), and perhaps the amount of ray parenchyma at the tissue level (Olano et al., 2013). There are some hypotheses suggesting that coordinated traits combined – not one trait alone – determines the safety/efficiency requirements and ultimately the species distribution (Hacke, 2015; Carlquist, 2017). Recent physiological and anatomical studies on bordered pits provide new insights into the mechanisms and evolution of conifer’s drought resistance (Choat et al., 2008; Hacke, 2015). However, the eco-physiological functions and evolution behind earlywood and latewood tracheids has been overlooked. We postulated that tracheid dimensional differences between earlywood and latewood have evolved to allow conifers to adapt to various environmental conditions, especially freezing conditions and drought.

Resistance to embolism is a crucial trait in trees to cope with cold and drought stresses, which further determines the geographic distribution of conifer species (Choat et al., 2012). Embolism resistance might be influenced by the structure and function of bordered pits and tracheids in xylem as well (Losso et al., 2018). A small alteration in xylem anatomy can lead to different performances in terms of water transport efficiency, embolism resistance and hydraulic capacitance (Hacke, 2015). Our phylogenetic analyses provide some evidence for functional optimization of xylem structures for species from different clades (Figures 4, 6). However, due to a currently incomplete understanding of embolism in tracheids, it is unclear whether anatomical traits such as tracheid dimensions, pit structure (Roskilly et al., 2019) and amount of axial parenchyma (Olano et al., 2013), evolved in parallel during conifer evolution, making it an interesting topic for further study.

## Conclusion

In this comparative study, variance of conifer xylem structure provides further evidence of conifer adaptation to various environmental conditions. In a latitudinal range from 18° N to 54° N, tracheid dimensions of 78 conifer species showed a consistent xylem pattern, where approximately half of the variance was found to be attributable to climatic and soil factors in tandem with phylogeny. The strong xylem structural divergence along phylogeny in Pinaceae species in contrast to Cupressaceae, Taxaceae and Cupressaceae further suggest that tracheid dimensions are an evolutionary outcome for conifers adapting to cold and drought stresses. Further work on the interspecific variability of conifer xylem would be required to better understanding hydraulic traits and functional adaptation to different environment for conifers. Given the fast development of image processing techniques and automatic climate and soil monitoring methods, we believe that a combination of information from radial growth, wood density and xylem anatomy will open up a new research area for tree-ring science and allows for the detection of large structural, biogeographical and environmental patterns as well as their interpretation, providing valuable references for future forest management under climate change.

## Data Availability Statement

The original contributions presented in the study are included in the article/Supplementary Material, further inquiries can be directed to the corresponding author/s.

## Author Contributions

JZ conceived the study. YL and JZ collected the data and did the statistical analyses. JZ, HM, FV, and SJ wrote the manuscript. All authors contributed to the article and approved the submitted version.

## Conflict of Interest

The authors declare that the research was conducted in the absence of any commercial or financial relationships that could be construed as a potential conflict of interest.

## Publisher’s Note

All claims expressed in this article are solely those of the authors and do not necessarily represent those of their affiliated organizations, or those of the publisher, the editors and the reviewers. Any product that may be evaluated in this article, or claim that may be made by its manufacturer, is not guaranteed or endorsed by the publisher.

## Footnotes

1
http://www.theplantlist.org/

2
http://srtm.csi.cgiar.org

3
http://www.worldclim.com/version2/

4
https://soilgrids.org/
