# Supplementary material for: Variation in Tracheid Dimensions of Conifer Xylem Reveals Evidence of Adaptation to Environmental Conditions
Source: Front Plant Sci. 2022 Feb 17;13:774241. doi: 10.3389/fpls.2022.774241 (PMC8893226; doi:10.3389/fpls.2022.774241)

Supplementary Material

***Variation of tracheid dimensions in conifer xylem reveals evidence of adaptation to environmental conditions***

**Jingming Zheng^1*^, Yajin Li^1^, Hugh Morris^2^, Filip Vandelook^3^, Steven Jansen^4^**

*** Correspondence:** Jingming Zheng: zhengjm@bjfu.edu.cn

**Appendices legend**

**Table S1.** Traits data examined in this study. H_max_, maximum plant height; CTD: tracheid tangential diameter; CRD: tracheid radial diameter; CL: tracheid length; WTT: tracheid wall tangential thickness; WRT: tracheid wall radial thickness; BPT: structural type of bordered pit. Subscript “e” stands for earlywood and “l” for latewood. Data in the last four columns, i.e., average diameter for earlywood and latewood (ADE, ADL), average wall thickness for earlywood and latewood (AWTE, AWTL), were from other sources for reference.

**Table S2** Paired T-test of tracheid traits from earlywood and latewood with and without taking phylogeny into account

**Table S3.** Univariate phylogenetic models for tracheid traits as functions of environmental variables and maximum plant height. ‘*’, ‘**’, “***” and ‘NS’ indicate p<0.001, p<0.01, p<0.05, and p>0.05 for the model, respectively. Values in bracelet stands for R^2^ contributed by environmental variables alone, which are calculated by the method of Ives (2019). CTD: tracheid tangential diameter; CRD: tracheid radial diameter; CL: tracheid length; WTT: tracheid wall tangential thickness; WRT: tracheid wall radial thickness. Subscript “e” stands for earlywood and “l” for latewood. Hmax: plant maximum height; LON: midpoint longitude; LAT: midpoint latitude; ALT: midpoint altitude; MAT: mean annual temperature; TSEA: temperature seasonality; MAP: mean annual precipitation; PSEA: precipitation seasonality; PH: soil pH; CFVO: coarse fragments (volumetric); SAND: sand content; SILT: silt content; CLAY: clay content.

**Table S4.** Top PGLS models of xylem tracheid characters as functions of climatic and soil variables, with models’ R^2^ for phylogeny and environmental variables partitioned by the method of Ives (2019). CTD: tracheid tangential diameter; CRD: tracheid radial diameter; CL: tracheid length; WTT: tracheid wall tangential thickness; WRT: tracheid wall radial thickness. Subscript “e” stands for earlywood and “l” for latewood. MAT: mean annual temperature; TSEA: temperature seasonality; MAP: mean annual precipitation; PH: soil pH; SILT: silt content; CLAY: clay content. The most parsimonious model was selected based on lowest AICs for each trait if delta-AIC between two corresponding models for the same trait is less than 2.

**Figure S1.** Illustration of tracheid traits measured in the cross section of a wood slide for a hypothetical conifer species.

**Figure S2.** Heat plot for correlations among environmental variables under a monsoonal climate in China. LAT=mid-point latitude, LON=mid-point longitude, ALT=mid-point altitude, MAT=mean annual temperature, TSEA=temperature seasonality; MAP=mean annual precipitation, PSEA= precipitation seasonality; PH=soil pH; CFVO= coarse fragments (volumetric); SAND=sand content; SILT=silt content; CLAY=clay content. Legend color bars represent the value of r (Pearson correlation coefficients), “*”, “**” and “***” stands for p<0.05, p<0.01, and p<0.001, respectively.

**Table S1.** Traits data examined in this study. H_max_, maximum plant height; CTD: tracheid tangential diameter; CRD: tracheid radial diameter; CL: tracheid length; WTT: tracheid wall tangential thickness; WRT: tracheid wall radial thickness. Subscript “e” stands for earlywood and “l” for latewood. Data in the last four columns, i.e., average diameter for earlywood and latewood (ADE, ADL), average wall thickness for earlywood and latewood (AWTE, AWTL), were from other sources for reference.

| Species | Family | H_max_  (m) | CTD.e  (µm) | CTD.l  (µm) | CRD.e  (µm) | CRD.l  (µm) | WTT.e  (µm) | WTT.l  (µm) | WRT.e  (µm) | WRT.l  (µm) | ADE  (µm) | ADL  (µm) | AWTE  (µm) | AWTL  (µm) |
| --- | --- | --- | --- | --- | --- | --- | --- | --- | --- | --- | --- | --- | --- | --- |
| *Abies delavayi* | Pinaceae | 50 | 40 | 39 | 46 | 19 | 2.5 | 3.3 | 2.5 | 4.0 |  |  |  |  |
| *Abies fabri* | Pinaceae | 30 | 34 | 31 | 45 | 18 | 2.1 | 3.6 | 2.1 | 3.8 | 38 | 35 | 3.8 | 4.9 |
| *Abies fargesii* | Pinaceae | 25 | 31 | 30 | 36 | 23 | 3.4 | 4.3 | 3.3 | 4.4 | 27 | 27 | 2.8 | 3.1 |
| *Abies forrestii* | Pinaceae | 35 | 38 | 35 | 44 | 24 | 3.2 | 5.4 | 3.0 | 6.0 | 38 | 35 | 3.2 | 5.4 |
| *Abies holophylla* | Pinaceae | 30 | 36 | 36 | 43 | 21 | 2.4 | 4.0 | 2.2 | 4.4 | 38 | 32 | 2.8 | 4.4 |
| *Abies nephrolepis* | Pinaceae | 30 | 32 | 31 | 48 | 24 | 2.9 | 4.4 | 2.7 | 4.2 | 34 | 31 | 3.2 | 3.8 |
| *Abies recurvata* | Pinaceae | 17 | 28 | 31 | 47 | 21 | 2.5 | 4.4 | 2.4 | 4.1 |  |  |  |  |
| *Abies sibirica* | Pinaceae | 40 | 36 | 32 | 48 | 24 | 3.2 | 4.8 | 3.8 | 5.1 | 36 | 32 | 3.2 | 4.8 |
| *Amentotaxus argotaenia* | Taxaceae | 20 | 30 | 30 | 32 | 25 | 4.4 | 4.0 | 3.9 | 4.0 | 29 | 30 | 5.1 | 5.0 |
| *Calocedrus formosana* | Cupressaceae | 12 | 37 | 39 | 39 | 23 | 3.9 | 5.1 | 3.9 | 6.1 |  |  |  |  |
| *Calocedrus macrolepis* | Cupressaceae | 10 | 34 | 30 | 35 | 19 | 2.9 | 3.5 | 2.9 | 3.6 | 26 | 26 | 3.1 | 3.5 |
| *Cathaya argyrophylla* | Pinaceae | 15 | 33 | 30 | 40 | 21 | 2.4 | 4.0 | 2.2 | 5.5 | 35 | 31 | 4.0 | 6.6 |
| *Cephalotaxus fortunei* | Taxaceae | 50 | 27 | 26 | 27 | 24 | 3.8 | 4.2 | 3.2 | 3.9 | 26 | 23 | 3.8 | 4.1 |
| *Cephalotaxus sinensis* | Taxaceae | 40 | 33 | 30 | 34 | 19 | 3.1 | 3.1 | 3.0 | 3.6 | 30 | 25 | 3.4 | 3.4 |
| *Chamaecyparis formosensis* | Cupressaceae | 30 | 47 | 46 | 47 | 21 | 2.6 | 4.5 | 2.7 | 5.3 | 37 | 37 | 3.4 | 4.2 |
| *Chamaecyparis obtusa* | Cupressaceae | 35 | 31 | 33 | 38 | 22 | 3.4 | 4.2 | 4.0 | 5.2 | 27 | 26 | 2.5 | 3.9 |
| *Cryptomeria japonica* | Cupressaceae | 25 | 31 | 30 | 39 | 21 | 2.5 | 4.2 | 4.3 | 4.3 |  |  |  |  |
| *Cunninghamia konishii* | Cupressaceae | 50 | 38 | 36 | 46 | 23 | 3.5 | 5.4 | 3.1 | 5.9 |  |  |  |  |
| *Cunninghamia lanceolata* | Cupressaceae | 40 | 37 | 34 | 41 | 23 | 2.8 | 4.3 | 2.8 | 4.4 | 31 | 30 | 27 | 51 |
| *Cupressus duclouxiana* | Cupressaceae | 35 | 26 | 25 | 27 | 17 | 3.0 | 3.0 | 2.7 | 3.4 | 27 | 25 | 4.1 | 4.5 |
| *Cupressus funebris* | Cupressaceae | 20 | 33 | 30 | 38 | 24 | 4.0 | 4.5 | 3.7 | 4.6 | 29 | 29 | 3.3 | 5.1 |
| *Dacrycarpus imbricatus* | Podocarpaceae | 30 | 38 | 34 | 42 | 21 | 3.8 | 3.8 | 4.1 | 4.1 |  |  |  |  |
| *Dacrydium pectinatum* | Podocarpaceae | 45 | 35 | 35 | 36 | 26 | 4.3 | 4.3 | 4.3 | 4.8 |  |  |  |  |
| *Fokienia hodginsii* | Cupressaceae | 30 | 41 | 38 | 46 | 30 | 3.2 | 5.9 | 3.4 | 5.4 | 36 | 30 | 3.8 | 4.4 |
| *Ginkgo biloba* | Ginkgoaceae | 50 | 35 | 34 | 35 | 24 | 3.2 | 3.9 | 3.7 | 4.2 | 36 | 33 | 3.1 | 3.2 |
| *Juniperus chinensis* | Cupressaceae | 30 | 25 | 24 | 29 | 20 | 3.2 | 3.4 | 2.8 | 3.4 | 28 | 25 | 4.0 | 4.7 |
| *Juniperus formosana* | Cupressaceae | 50 | 23 | 23 | 21 | 13 | 2.1 | 2.3 | 2.3 | 2.8 | 21 | 22 | 3.0 | 3.1 |
| *Juniperus rigida* | Cupressaceae | 50 | 24 | 23 | 23 | 15 | 2.4 | 2.5 | 2.3 | 2.5 | 24 | 23 | 2.4 | 2.5 |
| *Juniperus saltuaria* | Cupressaceae | 15 | 20 | 23 | 24 | 16 | 2.1 | 2.7 | 2.6 | 3.1 | 20 | 17 | 2.3 | 2.8 |
| *Keteleeria davidiana* | Pinaceae | 50 | 38 | 35 | 53 | 27 | 2.4 | 6.2 | 2.4 | 6.6 | 43 | 31 | 2.3 | 4.8 |
| *Keteleeria evelyniana* | Pinaceae | 40 | 49 | 49 | 56 | 29 | 2.7 | 6.3 | 2.9 | 6.9 | 40 | 39 | 3.7 | 7.1 |
| *Keteleeria fortunei* | Pinaceae | 60 | 45 | 42 | 54 | 29 | 3.1 | 5.7 | 3.5 | 6.1 | 45 | 38 | 4.1 | 7.7 |
| *Larix gmelinii* | Pinaceae | 50 | 44 | 42 | 67 | 35 | 2.3 | 6.2 | 2.1 | 6.2 | 40 | 36 | 2.8 | 8.1 |
| *Larix griffithii* | Pinaceae | 35 | 46 | 43 | 61 | 23 | 2.3 | 6.0 | 2.3 | 5.8 |  |  |  |  |
| *Larix mastersiana* | Pinaceae | 30 | 37 | 35 | 54 | 20 | 2.2 | 6.1 | 2.1 | 5.5 | 46 | 43 | 2.3 | 6.0 |
| *Larix potaninii* | Pinaceae | 30 | 33 | 33 | 60 | 26 | 3.0 | 8.7 | 2.6 | 7.5 | 43 | 29 | 3.9 | 5.1 |
| *Larix sibirica* | Pinaceae | 30 | 49 | 44 | 55 | 26 | 2.6 | 5.2 | 2.6 | 6.0 | 34 | 25 | 3.6 | 6.0 |
| *Metasequoia glyptostroboides* | Cupressaceae | 30 | 33 | 29 | 38 | 23 | 3.2 | 4.2 | 3.5 | 4.5 | 43 | 32 | 3.1 | 5.4 |
| *Nageia nagi* | Podocarpaceae | 50 | 37 | 35 | 48 | 23 | 2.3 | 5.0 | 2.3 | 5.6 | 27 | 33 | 3.5 | 4.1 |
| *Picea asperata* | Pinaceae | 30 | 35 | 33 | 37 | 21 | 2.3 | 4.0 | 2.3 | 4.7 | 37 | 35 | 2.5 | 6.2 |
| *Picea brachytyla* | Pinaceae | 30 | 34 | 30 | 45 | 18 | 2.2 | 3.5 | 2.0 | 4.5 | 34 | 35 | 2.8 | 5.7 |
| *Picea jezoensis* | Pinaceae | 45 | 36 | 32 | 41 | 19 | 2.3 | 3.7 | 2.3 | 4.3 | 33 | 38 | 3.5 | 4.2 |
| *Picea koraiensis* | Pinaceae | 30 | 37 | 33 | 44 | 21 | 2.3 | 4.5 | 2.3 | 5.0 | 33 | 24 | 2.7 | 3.5 |
| *Picea likiangensis* | Pinaceae | 55 | 33 | 31 | 53 | 24 | 3.2 | 5.5 | 2.7 | 5.7 | 34 | 33 | 2.7 | 3.0 |
| *Picea morrisonicola* | Pinaceae | 35 | 44 | 39 | 47 | 28 | 2.5 | 6.3 | 2.2 | 6.8 | 30 | 38 | 2.5 | 3.7 |
| *Picea neoveitchii* | Pinaceae | 25 | 40 | 35 | 48 | 25 | 2.3 | 5.2 | 2.2 | 5.8 |  |  |  |  |
| *Picea purpurea* | Pinaceae | 25 | 39 | 36 | 48 | 24 | 2.2 | 4.8 | 2.1 | 5.5 | 38 | 33 | 2.7 | 6.4 |
| *Picea schrenkiana* | Pinaceae | 30 | 35 | 34 | 33 | 21 | 2.0 | 3.9 | 2.0 | 4.4 |  |  |  |  |
| *Picea smithiana* | Pinaceae | 70 | 41 | 36 | 41 | 22 | 2.4 | 4.5 | 2.6 | 5.3 | 30 | 27 | 2.4 | 3.1 |
| *Picea wilsonii* | Pinaceae | 30 | 38 | 33 | 38 | 27 | 2.6 | 5.5 | 3.1 | 6.5 |  |  |  |  |
| *Pinus armandii* | Pinaceae | 20 | 47 | 43 | 53 | 23 | 3.8 | 4.3 | 3.3 | 5.0 |  |  |  |  |
| *Pinus bungeana* | Pinaceae | 20 | 30 | 27 | 35 | 18 | 2.5 | 2.8 | 2.3 | 3.7 | 33 | 31 | 2.8 | 4.2 |
| *Pinus densata* | Pinaceae | 25 | 42 | 39 | 46 | 22 | 2.5 | 4.3 | 2.4 | 4.9 | 30 | 26 | 3.0 | 4.4 |
| *Pinus densiflora* | Pinaceae | 40 | 42 | 40 | 50 | 26 | 2.8 | 5.5 | 2.4 | 5.8 | 41 | 36 | 3.1 | 6.0 |
| *Pinus kesiya* | Pinaceae | 50 | 52 | 49 | 52 | 29 | 3.4 | 7.1 | 3.1 | 8.8 | 35 | 31 | 3.6 | 6.2 |
| *Pinus koraiensis* | Pinaceae | 75 | 42 | 39 | 57 | 23 | 3.1 | 4.4 | 2.9 | 5.5 | 34 | 30 | 2.9 | 3.9 |
| *Pinus kwangtungensis* | Pinaceae | 20 | 45 | 43 | 57 | 30 | 3.3 | 6.3 | 3.1 | 5.8 | 38 | 35 | 3.1 | 5.4 |
| *Pinus latteri* | Pinaceae | 30 | 44 | 42 | 57 | 29 | 3.3 | 6.7 | 4.0 | 7.0 | 47 | 40 | 3.5 | 6.6 |
| *Pinus massoniana* | Pinaceae | 25 | 45 | 42 | 58 | 29 | 3.0 | 6.2 | 2.5 | 5.9 | 53 | 44 | 3.7 | 6.8 |
| *Pinus morrisonicola* | Pinaceae | 50 | 41 | 39 | 46 | 24 | 3.4 | 5.3 | 3.3 | 6.2 |  |  |  |  |
| *Pinus roxburghii* | Pinaceae | 40 | 51 | 44 | 60 | 29 | 3.1 | 5.6 | 3.3 | 6.0 | 39 | 34 | 4.1 | 6.3 |
| *Pinus sibirica* | Pinaceae | 30 | 35 | 30 | 44 | 15 | 2.3 | 2.5 | 2.1 | 2.7 | 35 | 30 | 2.3 | 2.5 |
| *Pinus sylvestris* | Pinaceae | 50 | 41 | 39 | 44 | 20 | 2.0 | 3.5 | 2.0 | 5.3 | 34 | 32 | 2.0 | 3.2 |
| *Pinus tabuliformis* | Pinaceae | 30 | 44 | 41 | 47 | 24 | 2.6 | 4.6 | 2.6 | 5.4 |  |  |  |  |
| *Pinus taiwanensis* | Pinaceae | 25 | 41 | 39 | 47 | 24 | 2.6 | 5.4 | 2.4 | 6.2 |  |  |  |  |
| *Pinus wallichiana* | Pinaceae | 35 | 42 | 38 | 51 | 20 | 3.3 | 4.1 | 2.8 | 5.2 |  |  |  |  |
| *Pinus yunnanensis* | Pinaceae | 30 | 37 | 33 | 37 | 26 | 2.8 | 6.1 | 3.1 | 6.2 |  |  |  |  |
| *Platycladus orientalis* | Cupressaceae | 30 | 26 | 24 | 27 | 18 | 2.2 | 3.4 | 2.2 | 3.1 | 27 | 27 | 3.7 | 3.8 |
| *Podocarpus macrophyllus* | Podocarpaceae | 17 | 25 | 26 | 31 | 18 | 2.6 | 2.9 | 2.7 | 3.1 | 25 | 24 | 2.5 | 2.8 |
| *Podocarpus neriifolius* | Podocarpaceae | 40 | 27 | 27 | 30 | 24 | 2.8 | 3.8 | 3.1 | 4.1 |  |  |  |  |
| *Pseudolarix amabilis* | Pinaceae | 20 | 43 | 39 | 64 | 29 | 2.3 | 6.5 | 2.2 | 6.0 | 45 | 43 | 4.5 | 7.8 |
| *Pseudotsuga sinensis* | Pinaceae | 12 | 48 | 45 | 50 | 25 | 2.1 | 5.9 | 2.3 | 7.2 | 45 | 35 | 3.5 | 7.6 |
| *Taiwania cryptomerioides* | Cupressaceae | 10 | 54 | 48 | 67 | 27 | 3.9 | 6.3 | 3.8 | 8.1 |  |  |  |  |
| *Taxus cuspidata* | Taxaceae | 15 | 33 | 30 | 43 | 21 | 2.3 | 4.6 | 2.4 | 4.5 | 33 | 30 | 2.3 | 4.6 |
| *Taxus wallichiana* | Taxaceae | 50 | 29 | 28 | 30 | 23 | 2.8 | 3.6 | 3.0 | 4.5 | 20 | 19 | 4.1 | 4.7 |
| *Torreya grandis* | Taxaceae | 40 | 33 | 32 | 36 | 24 | 3.6 | 4.4 | 3.0 | 4.7 | 31 | 31 | 2.8 | 4.7 |
| *Tsuga chinensis* | Pinaceae | 30 | 34 | 32 | 45 | 22 | 1.9 | 4.7 | 2.0 | 5.1 | 45 | 36 | 3.9 | 5.7 |
| *Tsuga dumosa* | Pinaceae | 35 | 40 | 38 | 46 | 25 | 2.4 | 4.8 | 2.5 | 5.0 | 38 | 35 | 2.8 | 5.4 |
| *Tsuga longibracteata* | Pinaceae | 25 | 37 | 35 | 48 | 23 | 2.3 | 5.0 | 2.3 | 5.6 | 40 | 33 | 2.9 | 6.6 |

**Table S2** Paired T-test of tracheid traits from earlywood and latewood with and without taking phylogeny into account

|  | Trait value (mean+sd) | | Paired T-test | | | Phylogenetic Paired T-test | | | |
| --- | --- | --- | --- | --- | --- | --- | --- | --- | --- |
|  | earlywood | latewood | t | df | p | t | df | p | lambda |
| Tracheid tangential diameter | 37.04 (0.92) | 34.78 (0.75) | 10.48 | 78 | <0.001 | 2.32 | 76 | 0.02 | 0.37 |
| Tracheid radial diameter | 44.00 (1.05) | 23.06 (0.95) | 22.51 | 78 | <0.001 | 2.05 | 76 | 0.04 | 0.85 |
| Tracheid tangential wall thickness | 2.81 (0.89) | 4.67 (1.37) | -13.10 | 78 | <0.001 | -0.91 | 76 | 0.36 | 0.91 |
| Tracheid radial wall thickness | 3.80 (0.82) | 5.09 (1.24) | -15.39 | 78 | <0.001 | -1.07 | 76 | 0.29 | 0.89 |

**Table S3.** Univariate phylogenetic models for tracheid traits as functions of environmental variables and maximum plant height. ‘*’, ‘**’, “***” and ‘NS’ indicate p<0.001, p<0.01, p<0.05, and p>0.05 for the model, respectively. Values in bracelet stands for R^2^ contributed by environmental variables alone, which are calculated by the method of Ives (2019). CTD: tracheid tangential diameter; CRD: tracheid radial diameter; CL: tracheid length; WTT: tracheid wall tangential thickness; WRT: tracheid wall radial thickness. Subscript “e” stands for earlywood and “l” for latewood. Hmax: plant maximum height; LON: midpoint longitude; LAT: midpoint latitude; ALT: midpoint altitude; MAT: mean annual temperature; TSEA: temperature seasonality; MAP: mean annual precipitation; PSEA: precipitation seasonality; PH: soil pH; CFVO: coarse fragments (volumetric); SAND: sand content; SILT: silt content; CLAY: clay content.

|  | PC1 | PC2 | CTD.e  (µm) | CTD.l  (µm) | CRD.e  (µm) | CRD.l  (µm) | WTT.e  (µm) | WTT.l  (µm) | WRT.e  (µm) | WRT.l  (µm) |
| --- | --- | --- | --- | --- | --- | --- | --- | --- | --- | --- |
| Hmax(m) | NS | NS | NS | NS | NS | NS | NS | NS | NS | NS |
| LON (°) | NS | NS | NS | NS | NS | NS | NS | NS | NS | NS |
| LAT(°) | 0.49(0.10)*** | 0.35(0.11)** | NS | 0.39(0.04)* | NS | 0.21(0.09)** | 0.28(0.14)** | 0.43(0.18)*** | 0.19(0.19)** | 0.43(0.16)*** |
| ALT(m) | NS | NS | NS | NS | NS | NS | NS | NS | NS | NS |
| MAT(℃) | 0.49(0.11)** | 0.33(0.08)** | NS | 0.38(0.02)* | NS | 0.22(0.11)** | 0.18(0.18)** | 0.40(0.13)*** | 0.21(0.21)** | 0.41(0.13)*** |
| TSEA | 0.48(0.09)** | 0.35(0.12)** | NS | 0.39(0.05)* | NS | 0.17(0.05)* | 0.28(0.15)*** | 0.41(0.14)*** | 0.16(0.16)*** | 0.41(0.13)*** |
| MAP(mm) | 0.49(0.12)*** | NS | 0.40(0.01)* | 0.39(0.05)** | NS | 0.22(0.11)** | 0.21(0.06)* | 0.45(0.20)*** | 0.13(0.13)* | 0.41(0.14)*** |
| PSEA | NS | NS | NS | NS | NS | NS | NS | NS | 0.07(0.07)* | NS |
| PH | 0.47(0.08)** | NS | NS | NS | 0.50(0.05)* | NS | 0.21(0.21)* | 0.42(0.16)*** | NS | 0.41(0.13)*** |
| CFVO | NS | NS | NS | NS | NS | NS | NS | NS | NS | NS |
| SAND | NS | NS | NS | NS | NS | NS | NS | NS | NS | NS |
| SILT | 0.52(0.17)*** | 0.37(0.14)*** | 0.47(0.14)*** | 0.47(0.16)*** | 0.53(0.05)* | 0.19(0.07)** | 0.30(0.17)*** | 0.39(0.12)*** | 0.35(0.20)*** | 0.43(0.17)*** |
| CLAY | NS | NS | NS | NS | NS | 0.17(0.05)* | NS | 0.34(0.04)* | NS | 0.36(0.06)** |

**Table S4.** Top PGLS models of xylem tracheid characters as functions of climatic and soil variables, with models’ R^2^ for phylogeny and environmental variables partitioned by the method of Ives (2019). CTD: tracheid tangential diameter; CRD: tracheid radial diameter; CL: tracheid length; WTT: tracheid wall tangential thickness; WRT: tracheid wall radial thickness. Subscript “e” stands for earlywood and “l” for latewood. MAT: mean annual temperature; TSEA: temperature seasonality; MAP: mean annual precipitation; PH: soil pH; SILT: silt content; CLAY: clay content. If delta-AIC between two corresponding models for the same trait is less than 2 then both model listed.

|  |  | Earlywood model | | | | |  | Latewood model | | | | |
| --- | --- | --- | --- | --- | --- | --- | --- | --- | --- | --- | --- | --- |
| Trait | formula | R^2^_tot_ | R^2^_var_ | R^2^_phy_ | AIC | lambda | formula | R^2^_tot_ | R^2^_var_ | R^2^_phy_ | AIC | lambda |
| PC1 | PC1~gls(MAP+SILTt) | 0.56 | 0.23 | 0.43 | 304.82 | 0.91 | PC2~gls(MAT+SILT) | 0.40 | 0.19 | 0.27 | 248.35 | 0.69 |
|  | PC1~gls(MAT+PH+SILT) | 0.56 | 0.23 | 0.43 | 305.52 | 0.90 |  |  |  |  |  |  |
|  | PC1~gls(MAT+MAP+SILT) | 0.56 | 0.23 | 0.43 | 305.53 | 0.90 |  |  |  |  |  |  |
|  | PC1~gls(MAT+SILT) | 0.55 | 0.22 | 0.43 | 305.86 | 0.90 |  |  |  |  |  |  |
| CTD | CTD.e~gls(SILT) | 0.47 | 0.14 | 0.39 | 500.34 | 0.86 | CTD.l~gls(MAT+SILT) | 0.48 | 0.18 | 0.31 | 481.29 | 0.81 |
|  | CTD.e~gls(MAT+SILT) | 0.47 | 0.14 | 0.39 | 501.14 | 0.85 | CTD.l~gls(MAP+SILT) | 0.48 | 0.18 | 0.31 | 481.80 | 0.82 |
| CRD | CRD.e~gls(PH+SILT) | 0.55 | 0.10 | 0.50 | 545.86 | 0.93 | CRD.l~gls(TSEA+CLAY) | 0.21 | 0.10 | 0.13 | 437.06 | 0.51 |
| WTT | WTT.e~gls(MAT+SILT) | 0.34 | 0.22 | 0.16 | 121.55 | 0.76 | WTT.l~gls(MAP+SILT) | 0.48 | 0.25 | 0.31 | 224.75 | 0.83 |
|  |  |  |  |  |  |  | WTT.l~gls(MAT+MAP+SILT) | 0.49 | 0.26 | 0.31 | 225.18 | 0.84 |
| WRT | WRT.e~gls(MAT+SILT) | 0.32 | 0.32 | 0.00 | 124.47 | 0.00 | WRT.l~gls(MAT+SILT) | 0.52 | 0.30 | 0.32 | 219.24 | 0.85 |
|  | WRT.e~gls(MAT+TSEA+SILT) | 0.32 | 0.32 | 0.00 | 125.74 | 0.00 | WRT.l~gls(MAT+MAP+SILT) | 0.53 | 0.31 | 0.32 | 219.56 | 0.85 |
|  |  |  |  |  |  |  | WRT.a~gls(MAT+PH+SILT) | 0.52 | 0.30 | 0.32 | 220.02 | 0.85 |

**Figure S1.** Illustration of tracheid traits measured in the cross section of a wood slide for a hypothetical conifer species.


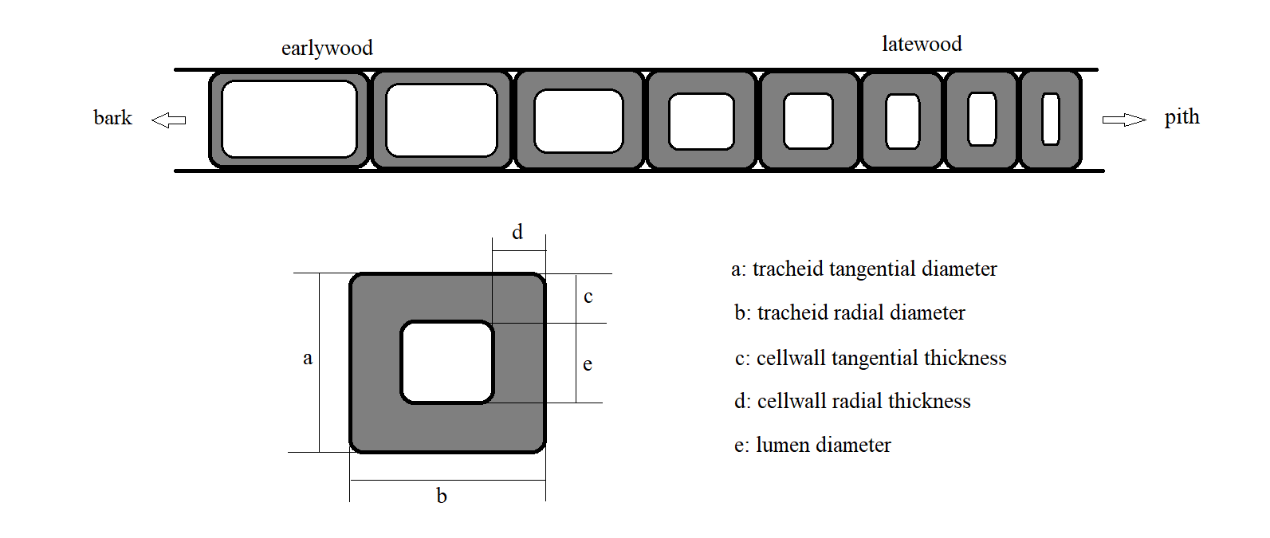


**Figure S2.** Heat plot for correlations among environmental variables under a monsoonal climate in China. LAT=mid-point latitude, LON=mid-point longitude, ALT=mid-point altitude, MAT=mean annual temperature, TSEA=temperature seasonality; MAP=mean annual precipitation, PSEA= precipitation seasonality; PH=soil pH; CFVO= coarse fragments (volumetric); SAND=sand content; SILT=silt content; CLAY=clay content. Legend color bars represent the value of r (Pearson correlation coefficients), “*”, “**” and “***” stands for p<0.05, p<0.01, and p<0.001, respectively.


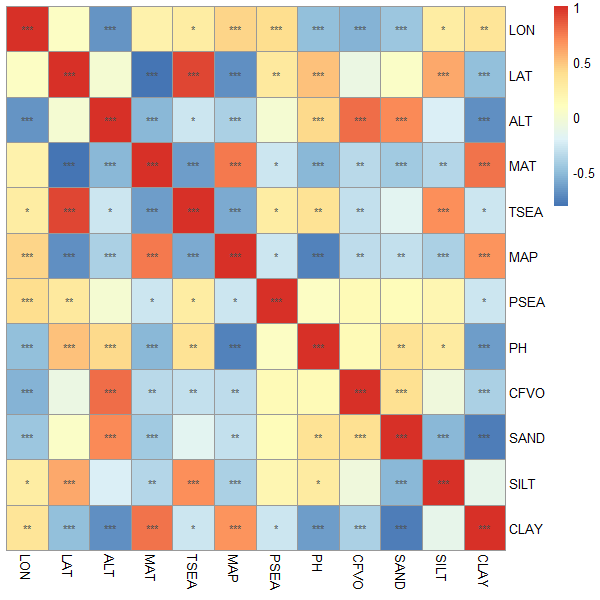

Supplement: Supplementary file 1 [file Data_Sheet_1.docx]
